# Disruption of Colorectal Cancer Network by Polyphyllins Reveals Pivotal Entities with Implications for Chemoimmunotherapy

**DOI:** 10.3390/biomedicines10030583

**Published:** 2022-03-02

**Authors:** Ram Siripuram, Zinka Bartolek, Ketki Patil, Saj S. Gill, S. Balakrishna Pai

**Affiliations:** Wallace H. Coulter Department of Biomedical Engineering, Georgia Institute of Technology and Emory University, Atlanta, GA 30332, USA; ram.siripuram@utah.edu (R.S.); zinkab@uw.edu (Z.B.); kpatil7@mail.gatech.edu (K.P.); sajindgill@gmail.com (S.S.G.)

**Keywords:** Polyphyllin D, Polyphyllin II, Polyphyllin G, DLD-1, HCT116, proteomics, apoptosis

## Abstract

The prevalence of colorectal cancer has increased world-wide with high rates of mortality and morbidity. In the absence of efficacious drugs to treat this neoplasia, there is an imminent need to discover molecules with multifaceted effects. To this end, we opted to study the effect of steroidal saponins such as Polyphyllins. We performed anticancer activity studies with three analogs of Polyphyllins: Polyphyllin D (PD), Polyphyllin II (PII) and Polyphyllin G (PG). Here we show the potent effect of PD, PII (IC_50_ of 0.5−1 µM) and PG (IC_50_ of 3 µM) in inhibiting the viability of colorectal adenocarcinoma cells (DLD-1) and colorectal carcinoma cells (HCT116). PD and PII also showed inhibition of cell proliferation and sustained response upon withdrawal of the compounds when assessed by clonogenic assays in both the cell lines. Elucidation of the molecular mode of action revealed impact on the programmed cell death pathway. Additionally, proteomic profiling of DLD-1 revealed pivotal proteins differentially regulated by PD and PII, including a downregulated peroxiredoxin-1 which is considered as one of the novel targets to combat colorectal cancers and an upregulated elongation factor 2 (EF2), one of the key molecules considered as a tumor associated antigen (TAA) in colon cancer. Entities of cell metabolic pathways including downregulation of the key enzyme Phosphoglycerate kinase 1 of the glycolytic pathway was also observed. Importantly, the fold changes per se of the key components has led to the loss of viability of the colorectal cancer cells. We envision that the multifaceted function of PD and PII against the proliferation of colorectal carcinoma cells could have potential for novel treatments such as chemoimmunotherapy for colorectal adenocarcinomas. Future studies to develop these compounds as potent anti-colorectal cancer agents are warranted.

## 1. Introduction

Among those cancers identified in the United States, colorectal cancer is the third most frequently encountered cancer with close to 104,270 new cases of colon cancer and 45,230 new cases of rectal cancer anticipated by 2021 [1]. Patients with localized cancers have a better survival rate up to 90% but the survival rate of patients with metastatic tumors is only 10%. Treatment of colorectal cancers depends on the stage of the disease and treatment modality involves surgical intervention to remove the tumor followed by chemotherapy and/or radiation therapy [2]. For colorectal cancers that have metastasized, the treatment is usually chemotherapy involving a combination of drugs such as: 5-fluorouracil, panitumumab, and irinotecan; either by themselves or in combination with monoclonal antibodies [2,3,4]. Recently there have been improvements in treatment strategies due to the use of newly discovered agents in combination with standard chemotherapeutics; in spite of these advanced treatments, clinical management has been more palliative than curative [5]. Therefore, there is an imminent need for novel, potent drugs with fewer side effects for the treatment of colorectal adenocarcinomas.

Naturally occurring plant derivatives have gained recognition as novel molecules against cancer and other diseases due to generally minimal side effects [6,7]. The herb *Paris polyphylla* has been studied for its anticancer properties against numerous cancers including neuroblastoma [8], lung cancer [9,10,11], cancer of the ovary [12], liver cancer [13], and human gastric carcinoma [14,15], human glioma [16] and myeloma [17]. *P. polyphylla* has also been shown to have antimicrobial effects [18]. *P. polyphylla* is rich in steroidal saponins such as polyphyllins, which are known to have medicinal properties [19]. Structurally, steroidal saponins are composed of a polysaccharide and a steroidal moiety, where both groups are required for the activity of the molecule [20]. Saponins as cytotoxic agents affect a large number of molecular pathways [21]. PG is known to induce apoptosis as well as autophagy in nasopharyngeal cancer cells via the activation of MAPK, ERK, AKT and JNK pathways [22]. Moreover, PG induced apoptosis and autophagy in human oral cancer cells [23] as well as G2/M cell cycle arrest in oral cancer (OECM-1) cells [18].

In the present study, we investigated whether steroidal saponins PD, PII, and PG exhibit an inhibitory effect on human colorectal cancer cells. To this end, we tested their effect on DLD-1 (human colorectal adenocarcinoma cell line) and HCT116 (a human colorectal carcinoma cell line). The investigation involved anticancer activity assessment to characterize the cellular inhibitory effects of PD, PII and PG and to elucidate the molecular mode of action by flow cytometry. We used 2-Dimensional Difference Gel Electrophoresis (2D-DIGE) and mass spectrometry, which are able to detect molecules differentially regulated at the femtomolar range, to identify key moieties of colon cancer pathways impacted by these molecules. Understanding the anticancer effects of PD, PII and PG would allow for development of novel treatment regimens for colorectal cancers.

## 2. Materials and Methods

### 2.1. Cell Culture and Reagents

Human colon cancer cell lines DLD-1 and HCT116 were obtained from American Type Culture Collection. DLD-1 cells were grown in RPMI-1640 supplemented with 10% FBS, 1% l-glutamine and 1% Penicillin-Streptomycin. HCT116 cells were grown in McCoy’s 5a supplemented with 10% FBS and 1% Penicillin-Streptomycin antibiotic solution. Cells were maintained at 37 °C under humidified conditions at 5% CO_2_. 20 mM stock solution of Polyphyllin D (PD), Polyphyllin II (PII) and Polyphyllin G (PG), all from ChemFaces, China (CFN90255, CFN99953, and CFN99955 respectively), were made in DMSO and stored at −20 °C. All the dilutions were made fresh in media.

### 2.2. Cytotoxicity Assays

Cytotoxicity assays were performed as described previously [24]. Briefly, cells were plated at a density of 5000 cells per well in a 96-well plate and incubated at 37 °C in an incubator with 5% CO_2_ and 95% air for 24 h. Varying concentrations of PD, PII and PG ranging from 0.1 µM to 100 µM were added in triplicates and incubated for 72 h along with the control sample. To assess the cytotoxicity, Cell Counting Kit-8 (CCK-8 kit, Bimake, China) was used and the absorbance at 450 nm was acquired on Tecan’s infinite 200 pro plate reader (Switzerland).

### 2.3. Clonogenic Assay

Clonogenic assays were performed as described previously [24]. At the end of the 72 h treatment period with 0.5 µM of either PD or PII, 500 cells per well were plated in triplicates in a 6 well plate along with a control group and incubated for 7–9 days without media change. To assess the colony forming efficiency of the cells upon treatment, cells were washed with PBS, fixed in methanol and stained with 0.1% crystal violet in 25% methanol for 10 min, after which the excess stain was removed by washing with water. For quantitative analysis of the colonies, cells were lysed with 1% SDS and the released crystal violet intensity was read at 595 nm using the plate reader.

### 2.4. Muse Cell Analysis

For quantitative molecular analysis, the protocol followed was as described [25]. Cells were plated at a density of 100,000 per well in 6 well plates and allowed to adhere for 24 h. The cells were treated with 0.5 µM of PD or PII individually in triplicates for 72 h. Controls with no treatments were maintained in parallel with the treatment groups. After 72 h, cells were harvested by trypsinization and prepared as per the Muse assay protocol for Annexin V and Dead cell (MCH100105). The stained cells were analyzed on Muse cell analyzer (Millipore, Burlington, MA, USA).

### 2.5. Proteomic Analysis

The protocol employed for proteomic analysis was as described previously [26]. Cells were plated per flask and after 24 h, media was changed for control group and for treatment groups growth medium was added with 0.5 µM of either PD or PII and incubated for 72 h. Cell pellets from duplicate flasks of untreated and treated cells were sent to Applied Biomics (Hayward, CA, USA) for proteomic analysis. Proteins from both groups were analyzed, utilizing 2D-DIGE for protein expression profiling. Protein spots that had greater than 1.5-fold increase or decrease in expression and differential expression were selected for matrix-assisted laser desorption/ionization-Time of Flight (MALDI-TOF) mass spectrometry (MS) for protein identification.

### 2.6. Statistical Analysis

All the toxicity and Muse assays were performed thrice independently and the data were normalized to control and then averaged. All the data were analyzed using GraphPad Prism 7 (San Diego, CA, USA) and results expressed as ± SD with *p* < 0.05 considered significant. One-way ANOVA followed by Dunnett’s multiple comparisons test was performed to assess the significant differences between control and all the treatment groups.

## 3. Results

### 3.1. Polyphyllin D (PD) and Its Structural Analogs PII and PG Inhibit the Viability of Colorectal Cancer Cells

**Figure 1:**
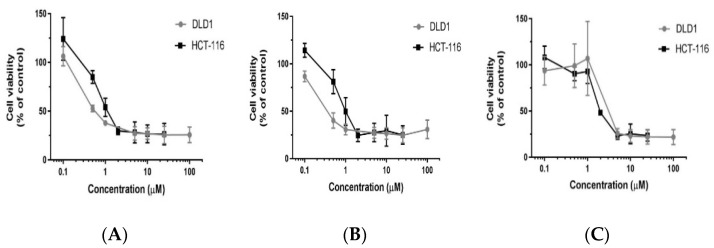
Cytotoxicity of PD, PII and PG on DLD-1 and HCT116 cells. Cells were plated in 96 well-plates at a density of 5000 cells per well. After 24 h, the cells were treated with various concentrations of PD (**A**), PII (**B**), and PG (**C**) ranging from 0.1 µM to 100 µM and incubated for 72 h in triplicates. A CCK-8 assay was performed to assess the cell viability. The data points are the mean of three such independent experiments. Percent of viable cells in treatment groups was calculated by considering untreated control values as 100% and there was a significant decrease in cell viability in all treatment groups as per one-way ANOVA when compared to the untreated group. The *p*-value for DLD-1 cells was <0.0001, when treated with PD and PII from 0.5 µM onwards and from 2 µM onwards when treated with PG. The *p*-value for HCT116 cells was <0.0001, when treated with PD and PII from 1 µM onwards and from 2 µM onwards when treated with PG.

To assess the potential of polyphyllin D and its structural analogs PII and PG (structural differences shown in Supplementary Figure S1) to inhibit the viability of colorectal cancers, cells from DLD-1 and HCT116 were treated with varying concentrations of the three compounds and incubated for 72 h. As the concentration of all the three compounds were increased, the viability decreased and a dose dependent response was observed for each of the compounds. The inhibition of viability was assessed by CCK-8 assay. Considering untreated control values as 100%, the percent of viable cells in the treatment groups were calculated. There was significant decrease in viability of DLD-1 as well as HCT116 cells when treated with PD as shown in Figure 1A. The IC_50_ was 0.5 µM for PD in DLD-1 and 1µM in HCT116. When the analog PII was tested in a similar manner in both the cell systems, a dose-dependent response was also observed (B), with an IC_50_ of 0.5 µM in DLD-1 and 1.5 µM in HCT116. Similarly, when the analog PG was administered, a dose-dependent response was also observed as shown in (C). The IC_50_ values were 3 µM in DLD-1 cells and 2 µM in HCT116. The effect of PG on both the cell lines was not as sensitive as that observed with PD and PII. Thus, further studies were performed with PD and PII.

### 3.2. Polyphyllin D (PD) and PII Impact the Colony Forming Efficiency of DLD-1 and HCT116 Cells Leading to a Sustained Response

**Figure 2:**
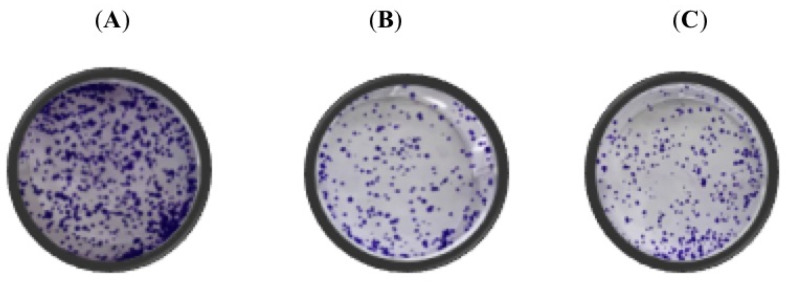

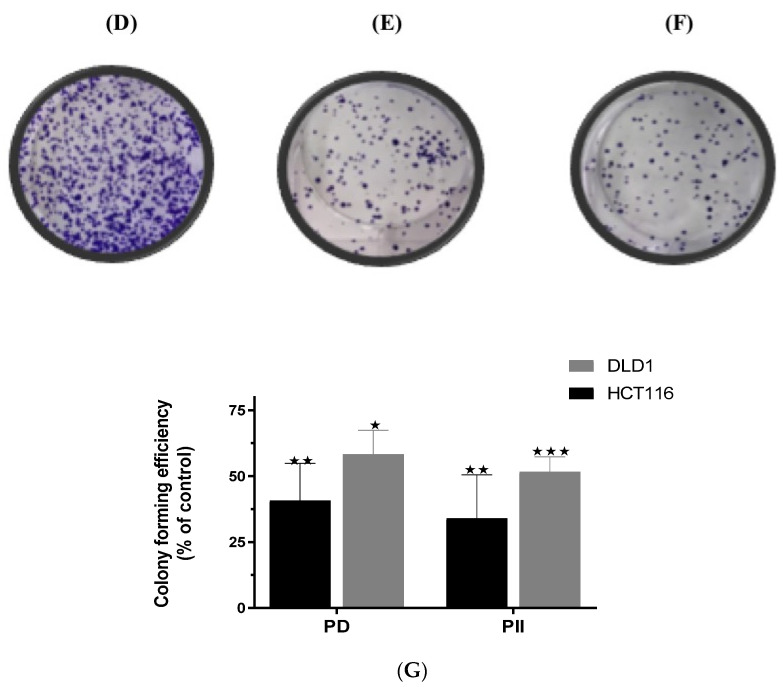
Colony forming efficiency of DLD-1 and HCT116 cells treated with PD and PII. Cells treated for 72 h with 0.5 µM of either PD or PII were seeded in 6 well-plates at a density of 500 cells per well in triplicates and allowed to form colonies over a period of 7–9 days. The colonies were fixed, and stained with crystal violet. For quantitative analysis, fixed colonies were lysed with 1% SDS, and absorbance readings were taken at 595 nm. The data points are the mean of three such independent trials. When the absorbance values of the treatment groups were normalized to the respective controls; there was a significant decrease in the colony forming efficiency of the treated cells when compared to control when assessed by one-way ANOVA. The *p*-value for DLD-1 cells was 0.03 when treated with PD and 0.0001 with PII treatment. For HCT116 cells, the *p*-value was <0.005 when treated with both PD and PII. (**A**) Representative image of clonogenic assay performed in DLD-1 control sample, (**B**) PD treated DLD-1sample, (**C**) PII treated DLD-1 sample. (**D**) Representative image of clonogenic assay performed in HCT116 control sample, (**E**) PD treated HCT116 sample, (**F**) PII treated HCT116 sample. (**G**) Colony forming efficiency of the treated samples normalized to the respective control samples are represented for both the cell systems. * *p*-value < 0.05; ** *p*-value < 0.01; *** *p*-value < 0.001.

Data from the effect of PD and PII on cell viability of colorectal cancer cells revealed that PD and PII exhibited potent inhibition at low concentration of 0.5 to 1 µM. To further assess their impact on proliferation, DLD-1 and HCT116 cells were individually treated with 0.5 µM of PD or PII for 72 h and then were sub-cultured in 6-well plates in drug-free medium and allowed to form colonies over a period of 7–9 days in the absence of the compounds. A representative image of the control sample is shown in Figure 2A, PD-treated sample (B), PII-treated sample (C). The colony-forming efficiency of DLD-1 cells was significantly impacted, as very few colonies were observed in the treatment group when compared to the control group. Data from HCT116 were similar to that observed in DLD-1 cells as shown in Figure 2D–F for the control group, the PD-treated group and the PII-treated group respectively. The inhibition of proliferation observed was sustained because the colonies were reduced, even on withdrawal of the compounds. For each of the cell lines, the data were normalized to the control group as shown in Figure 2G. The observation of a significant decrease in the number of colonies in both the cell systems reflects upon the impact of PD and PII on the entities governing the proliferation ability of the colorectal cancer cells.

### 3.3. Elucidation of the Mechanism of Action of PD and PII Revealed Impact on Programmed Cell Death Pathway in DLD-1 Cells

**Figure 3:**
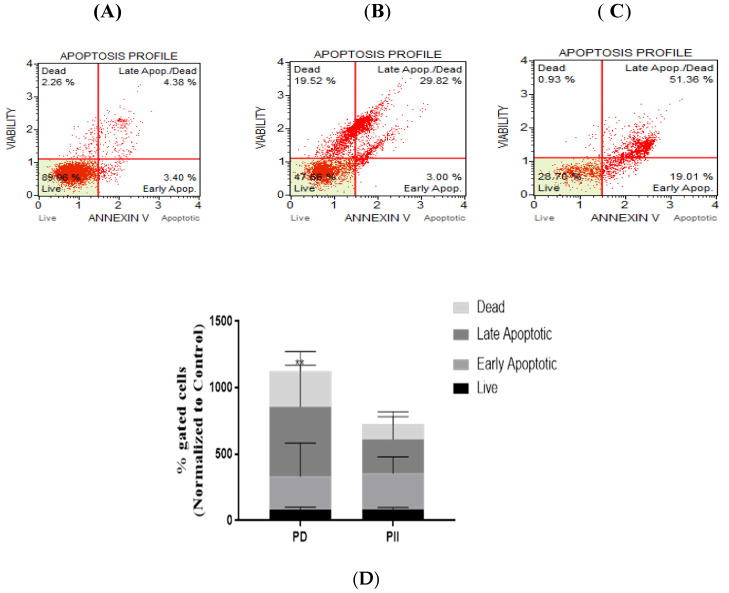
Analysis of apoptosis in DLD-1 cells treated with PD and PII. Representative scatter plots of three independent experiments performed for apoptotic population analysis by Annexin V detection. Cells treated with 0.5 µM of either PD or PII for 72 h were stained with the Muse Annexin V and Dead Cell reagent, and acquired on the Muse Cell Analyzer. Control sample (**A**), PD-treated sample (**B**), PII-treated sample (**C**), % gated profiles from the flow cytometry data normalized to the control for each of the treatments is shown (**D**), ** *p*-value = 0.0016.

On observing the potent impact of PD and PII on cell viability and cell proliferation, we proceeded to investigate the mechanism of action of PD and PII in causing cell death of DLD-1 cells. Studies were conducted to determine if induction of apoptotic pathway was activated. For this assessment, Annexin V Muse flow cytometric assay was performed. The representative profile of the control group is shown in Figure 3A, PD-treated group (B) and PII-treated group (C). On analyzing the data from the scatter plots, an increase in apoptotic cell population in the treated group was observed when compared to the control group. The % gated profiles from the flow cytometry data normalized to the control for each of the treatments is shown (D). The data observed indicates that apoptotic pathway was induced by PD and PII. PII-treated samples did not show a statistically significant increase, but on comparing the late apoptosis fraction of the control versus the PD-treated sample, a significant increase was observed with a *p*-value of 0.0016.

### 3.4. Studies on the Mechanism of Action of Polyphyllins PD and PII Revealed Activation of Programmed Cell Death Pathway in HCT116 Cells

**Figure 4:**
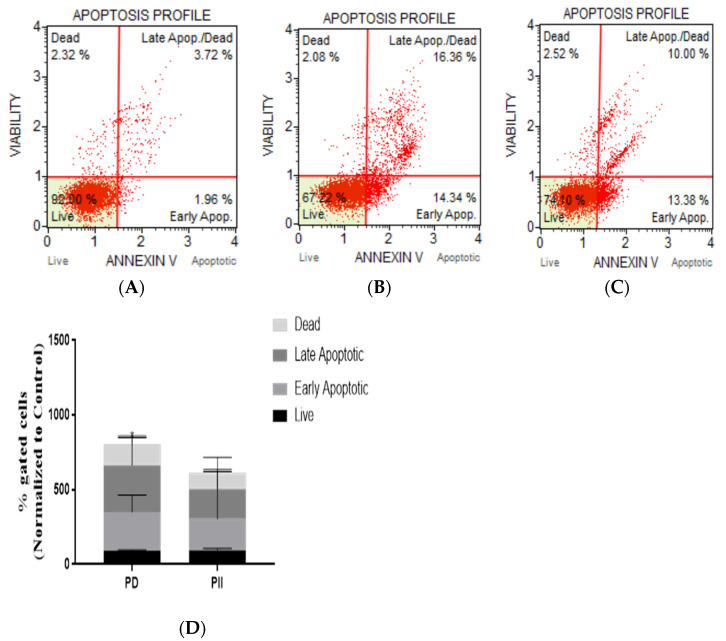
Analysis of apoptosis in HCT116 cells treated with PD and PII. Representative scatter plots of three independent experiments performed for apoptotic population analysis by Annexin V detection. Cells were treated with 0.5 µM of either PD or PII for 72 h, stained with the Muse Annexin V and Dead Cell reagent, and acquired on the Muse Cell Analyzer. Control sample (**A**), PD-treated sample (**B**), PII-treated sample (**C**), % gated profiles from the flow cytometry data normalized to the control for each of the treatments is shown (**D**), * *p*-value = 0.032.

Our studies on colorectal adenocarcinoma cells (DLD-1) revealed that the programmed cell death pathway is induced. Therefore, we opted to elucidate the mode of action of cell death caused by PD and PII in in colorectal carcinoma cells (HCT116). The analysis was performed using the Annexin V Muse flow cytometric assay. Cells were treated with 0.5 µM of each of the Polyphyllins and were incubated for 72 h and compared with the untreated (control) group. The profiles of the control group are shown in Figure 4A, PD-treated group (B), and the PII-treated sample (C). The change in population of each category of cells identified by flow cytometry on treatment with the two Polyphyllins when normalized to the control is shown in Figure 4D. In PII-treated cells, there was no statistically significant increase in the apoptotic cell population; whereas, in PD-treated cells, there was a significant increase in the late apoptotic cells when compared to the control sample with a *p*-value of 0.032.

### 3.5. Proteomic Analysis Revealed Key Entities of the Signaling Circuits in DLD-1 Cells That Were Differentially Regulated on Administration of PD and PII

**Figure 5:**
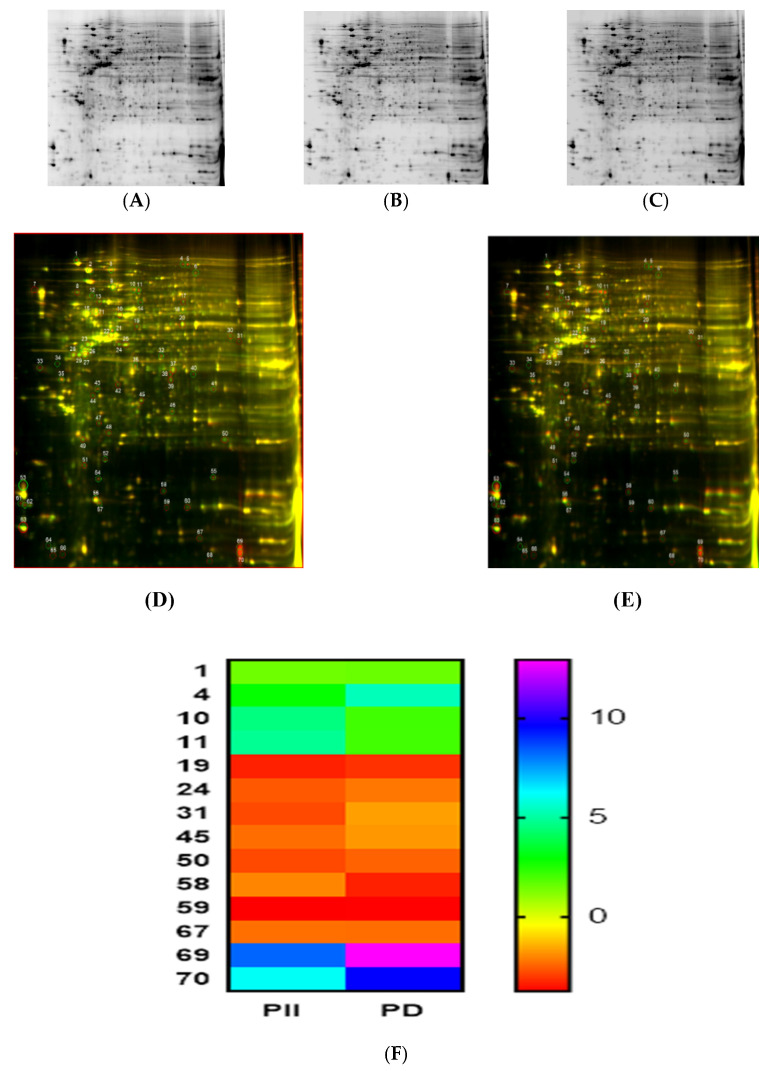
Proteomic analysis of DLD-1 cells treated with PD and PII. DLD-1 cells were treated with PD at 0.5 µM in duplicates of T-25 flask as mentioned in ‘Materials and Methods.’ Similarly, DLD-1 cells were treated with PII at 0.5 µM in duplicates and processed in an identical manner. In parallel, untreated DLD-1 cells (control group) were also processed in an identical manner. In (**A**), the 2D gel image is shown of the control (untreated) sample. 2D gel images of PD and PII treated samples are shown in (**B**,**C**). Gel image of an overlay of the control and PD-treated sample is shown in (**D**) and the PII-treated sample in (**E**). The number of differentially expressed proteins are shown in the heat map of proteins in (**F**) for PD and PII.

On observing the potent inhibition of cell viability and proliferation of DLD-1 cells by PD and PII, we opted to investigate further the impact on colorectal cancer’s signaling pathways by employing highly sensitive technologies such as 2D-DIGE and mass spectrometric analysis. DLD-1 untreated (control sample) and treated cells with PD, PII at the IC_50_ concentration were processed as described in ‘Materials and Methods’. Protein gel images of the control, PD- and PII-treated samples show clear separation of proteins (Figure 5A–C). Proteomic analysis was performed with the control proteins tagged with Cy2 (blue fluorescence), the PD-treated group with Cy3 (green fluorescence) and the PII-treated group with Cy5 (red fluorescence) prior to being subjected to electrophoresis. An overlay of the control and treated gels shows the differentially regulated proteins. Gel image of PD treated sample is shown in Figure 5D and PII-treated sample in Figure 5E. The number of differentially expressed proteins are shown in the heat map of proteins in Figure 5F for PD and PII.

Based on the fold changes observed, we selected upregulated and downregulated proteins that showed similar increase/decrease in levels in both PD- and PII-treated DLD-1 cells when compared to the controls for further proteomic analysis by mass spectrometry. The details of the molecules profiled are represented in Table 1. These key entities are components of pivotal cell physiological pathways; thus, their deregulation has impacted the viability of DLD-1 cells. Among the downregulated proteins was 26S proteasome regulatory subunit 7. The decrease in this molecule could have a limiting effect on the growth of colorectal cancer cells, as inhibitors of proteasomes are considered anticancer agents. Similarly, phosphoglycerate kinase 1 level was downregulated. Phosphoglycerate kinase 1 is considered as a promoter of metastasis in colon cancer. Therefore, its downregulation could prevent metastasis of colorectal adenocarcinomas. Another molecule downregulated was peroxiredoxin-1. Furthermore, ribosomal proteins like the 40S ribosomal protein S18 is known to promote cancer cell growth by inhibiting P27; polyphyllin’s action on decreasing this critical entity reflects upon the inhibition of cell growth of DLD-1 cells that we observed in the cellular level analysis. Furthermore, cell scaffold is controlled by keratin molecules. Keratin type 1 cytoskeletal was downregulated in the present study. This could have a major effect on the epidermis, the mitochondrial lipid composition and could have an effect on the mitochondrial activity itself in colorectal cancer cells. Furthermore, cofilin 1, which is involved in cytoskeletal remodeling, was decreased. The combination of decrease in keratin and cofilin could have profound inhibitory effect on colon cancer cells. Additionally, histidine triad nucleotide-binding protein 1-Profilin1, a key molecule in actin cytoskeletal remodeling, was downregulated as well; this impact of Polyphyllins could deter colon cancer cell’s invasion ability. Moreover, the General Transcription factor 11.1 level was decreased; thus, the control of cancer gene transcription in DLD-1 colon cancer cells could be impaired.

**Figure 6:**
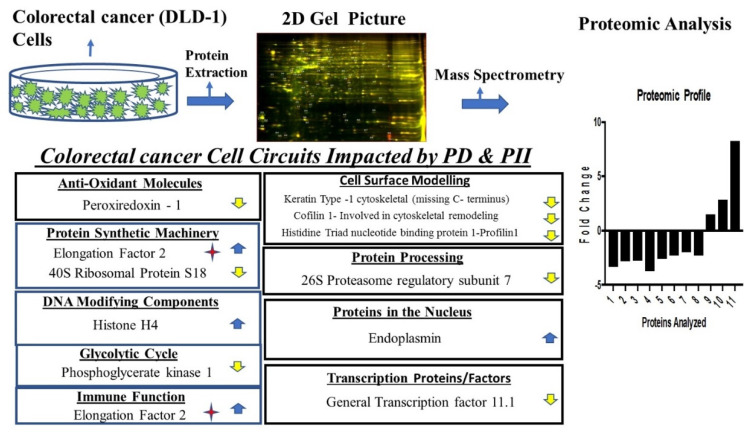
Schematic representation of pivotal pathways impacted by PD and PII in DLD-1 cells and the differentially regulated entities in each of the pathways identified. Arrows represent upregulated and downregulated molecules. The star represents its role in immune function. The numbers on the *x*-axis of the proteomic profile are the molecules numbered in the Table 1 for DLD-1.

Among the upregulated entities, the levels of Endoplasmin (heterogeneous nuclear riboprotein U-like protein) increased. This is in contrast to its downregulation in renal carcinoma. Similarly, elongation factor 2 (EF2) was upregulated by PD and PII. In colon cancers, EF2 is reported to elicit a cellular and humoral response and is a tumor-associated antigen (TAA). Therefore, its upregulation could aid in immunotherapy for colorectal cancers. Furthermore, histone H4 levels increased as well. An increase in this entity could lead to changes in chromatin, thus affecting gene expression of these cancer cells. A schematic network depicting the key cancer circuits of the colorectal adenocarcinoma cells impacted by PD and PII is shown in Figure 6.

## 4. Discussion

Our studies on anticancer drug discovery revealed that the structural analogs of Polyphyllin PD and PII have a potent inhibitory activity against colorectal cancer cells with IC_50_ as low as 0.5 µM in DLD-1 and 1 µM in HCT116, whereas another analog—PG—did not show potency at a low concentration similar to that of PD and PII. This study sheds light on the structure-activity of Polyphyllins, which are steroidal saponins with polysaccharide and steroidal moiety [19]. The steroidal saponins are known to affect a plethora of pathways in various cancer cells. We performed multilevel analysis to investigate the anticancer property of PD and PII in DLD-1 cells. We performed flow cytometry to elucidate the molecular mode of action of PD and PII, which revealed the impact of these compounds on the programmed cell death pathway. To delineate further the entities impacted in the DLD-1 cancer cell circuitry, we employed a highly sensitive methodology such as 2D-DIGE and mass spectrometry, which can identify differentially regulated molecules in the femtomolar-range [27].

A number of entities were downregulated as well as upregulated by PD and PII. Based on similar fold changes observed in PD- as well as PII-treated samples, we selected proteins in both the categories and have identified key regulators involved in the cancer circuitry of colorectal adenocarcinoma cells. One of the downregulated components was 26S proteasome regulatory subunit 7. A decrease in the expression of proteasomes have been reported during differentiation of hematopoietic cells [28]; furthermore, proteasome inhibitors that decrease proteasomes are considered as anticancer agents [29]. Moreover, a metabolic enzyme like the Phosphoglycerate kinase 1, a key regulator of the first ATP-generating step in glycolysis, was also downregulated. This kinase is reported to act as a promoter of colon cancer metastasis [30]. Thus, downregulation of this pivotal enzyme, essentially, could starve the colorectal cancer cells, and also inhibit metastasis of these cancer cells.

Peroxiredoxin-1 was another key molecule that was downregulated. Peroxiredoxin-1 is reported to promote metastasis and angiogenesis in colorectal cancers [31]. Thus, its downregulation by PD and PII could be beneficial in reducing metastasis and angiogenesis in colorectal cancers. Moreover, targeting peroxiredoxin-1 is considered as one of the novel strategies for treatment of colorectal cancers [32]. Recently it has been reported that, to overcome resistance to DNA-damaging agents like etoposide, targeting peroxiredoxin-1 could be a beneficial strategy [33]. As observed in the present study, action of Polyphyllins PD and PII in downregulating the key molecule peroxiredoxin-1 could be employed to achieve this novel treatment option.

Among the ribosomal proteins, 40S ribosomal Protein S18 was downregulated in the present study. Differential regulation of ribosomal proteins is reported in colorectal cancers [34]. Apart from the above-mentioned molecules, Keratin type 1 cytoskeletal–a keratin scaffold protein was downregulated. Keratin type 1 is shown to regulate cell architecture, cell proliferation, motility and apoptosis [35]; thus, its reduction would negatively impact colon cancer cells. Similarly, Cofilin 1, known to be involved in cytoskeletal remodeling, interacts with other proteins and is implicated in colon cancer cell migration [36]. Therefore, its decrease could reduce migration of colorectal cancers. Another important entity in this category is histidine triad nucleotide binding protein 1-(Profilin1) which is involved in cell migration as well [37]. The decrease in its levels by PD and PII would additionally reduce cell migration in colorectal cancers.

In the transcription factor category, the general transcription factor 11.1 is known to affect cancer circuitries [38]; therefore, its decrease by PD and PII could have an impact on the cancer transcriptional network.

Apart from the above-mentioned proteins that were downregulated by PD and PII, other entities were upregulated and they are: Endoplasmin (Heterogeneous nuclear riboprotein U-like protein), which is known to be downregulated in renal carcinoma and correlated with poor prognosis [39]. We envision that upregulation of this entity by PD and PII could have a beneficial effect on colorectal adenocarcinomas. Elongation Factor 2 has been reported to be a key molecule involved in translational control and is reported as a novel tumor associated antigen (TAA) in colon cancers [40]; therefore, its upregulation by PD and PII could aid in immune attack of colorectal cancers. Thus, a combination of agents such as Polyphyllins-PD, PII with immunotherapeutic agents could be a beneficial strategy in eliciting immune response against colorectal adenocarcinomas. In fact, it is reported that modified peptides derived from elongation factor 2 increased immune response to colon cancer [41]. Notably, saponins are reported to have properties of adjuvants that are used to enhance immune responses apart from having anticancer properties [42]. Furthermore, there are reports of modified saponins that could be harnessed for vaccine therapy in the clinic [43]; therefore, we envision that the multifaceted action of PD and PII could be harnessed as a novel chemoimmunotherapeutic strategy to combat colorectal cancers.

Among the chromatin modulators, Histone H4 was upregulated. Histone H4-acetylation and trimethylation loss is a key feature of cancer [44]. We envision that an increase in this entity leading to a chromatin level change affecting gene expression in colorectal cancer cells could lead to loss of viability, which we observed at the cellular level in the present study. Additionally, polyphyllins have been reported to inhibit cancer cells by varied mechanisms including reactive oxygen species (ROS) production and DNA damage [45].

In conclusion, the fold changes per se could determine the ultimate outcome observed, which is the decrease in cell viability of the colorectal adenocarcinoma cells. Therefore, a further development of compounds such as PD and PII, which exhibit potency at low concentrations and have a multifaceted impact on colorectal cancer cells including a potential to invoke immune response when administered as a chemoimmunotherapeutic is warranted.

## Figures and Tables

**Table 1:** Differentially Regulated Proteins on Impact by Polyphyllin D and PII in DLD-1 Cells.

SI #	Fold Change	Proteins Identified
	PII PD	
1	−3.33 −3.10	26S Proteasome regulatory subunit 7
2	−2.81 −1.67	Phosphoglycerate Kinase 1
3	−2.80 −2.46	Peroxiredoxin-1
4	−3.74 −3.73	40S Ribosomal Protein S18
5	−2.60 −2.20	Keratin type 1 cytoskeletal
6	−2.32 −1.77	General Transcription factor 11.1
7	−2.01 −3.33	Cofilin 1
8	−2.30 −2.33	Histidine triad nucleotide-binding protein 1-Profilin1
9	+1.48 +1.55	Endoplasmin
10	+2.88 +5.33	Elongation Factor 2
11	+8.28 +12.94	Histone H4

## Data Availability

All the data relating to this article are presented in the manuscript.

## Supplementary Materials

The following supporting information can be downloaded at: https://www.mdpi.com/article/10.3390/biomedicines10030583/s1, Figure S1: Chemical structures of Polyphyllin D, Polyphyllin G and Polyphyllin II.
