# Supplementary material for: Disruption of Colorectal Cancer Network by Polyphyllins Reveals Pivotal Entities with Implications for Chemoimmunotherapy
Source: Biomedicines. 2022 Mar 2;10(3):583. doi: 10.3390/biomedicines10030583 (PMC8945690; doi:10.3390/biomedicines10030583)
Supplement: Supplementary file 1 [file biomedicines-10-00583-s001.zip › Figure S1.pdf]

Figure S1

Polyphyllin D

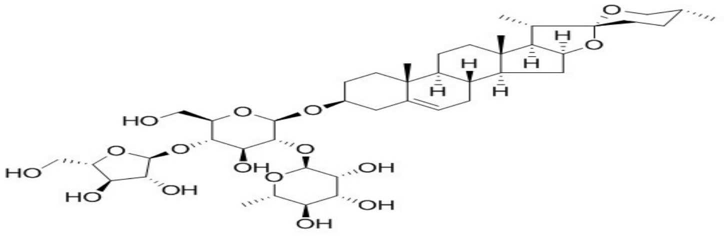

Polyphyllin G

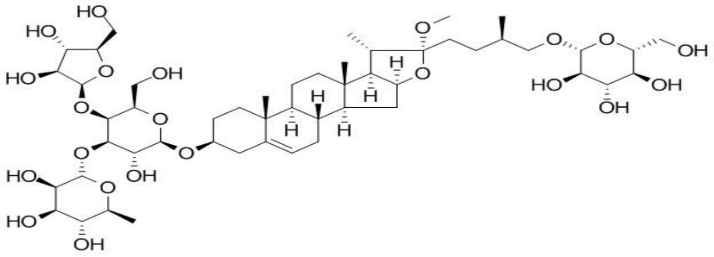

Polyphyllin II

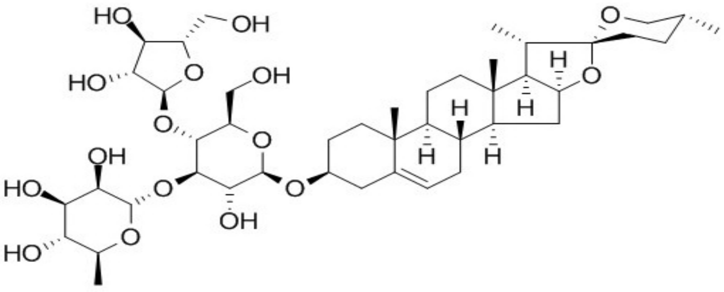

Figure S1. Chemical Structures of Polyphyllin D, Polyphyllin G and Polyphyllin II.
